# Genomic landscape of extended-spectrum β-lactamase resistance in *Escherichia coli* from an urban African setting

**DOI:** 10.1093/jac/dkx058

**Published:** 2017-03-04

**Authors:** Patrick Musicha, Nicholas A. Feasey, Amy K. Cain, Teemu Kallonen, Chrispin Chaguza, Chikondi Peno, Margaret Khonga, Sarah Thompson, Katherine J. Gray, Alison E. Mather, Robert S. Heyderman, Dean B. Everett, Nicholas R. Thomson, Chisomo L. Msefula

**Affiliations:** 1 Malawi-Liverpool-Wellcome Trust Clinical Research Programme, Queen Elizabeth Central Hospital, Blantyre, Malawi; 2 Microbiology Unit, Department of Pathology, College of Medicine, University of Malawi, Blantyre, Malawi; 3 Institute of Infection and Global Health, University of Liverpool, Liverpool, UK; 4 Liverpool School of Tropical Medicine, Liverpool, UK; 5 Wellcome Trust Sanger Institute, Wellcome Trust Genome Campus, Hinxton, UK; 6 Department of Veterinary Medicine, University of Cambridge, Cambridge, UK; 7 Division of Infection and Immunity, University College London, London, UK; 8 Department of Infectious and Tropical Diseases, London School of Hygiene and Tropical Medicine, London, UK

## Abstract

**Objectives:** Efforts to treat *Escherichia coli* infections are increasingly being compromised by the rapid, global spread of antimicrobial resistance (AMR). Whilst AMR in *E. coli* has been extensively investigated in resource-rich settings, in sub-Saharan Africa molecular patterns of AMR are not well described. In this study, we have begun to explore the population structure and molecular determinants of AMR amongst *E. coli* isolates from Malawi.

**Methods:** Ninety-four *E. coli* isolates from patients admitted to Queen’s Hospital, Malawi, were whole-genome sequenced. The isolates were selected on the basis of diversity of phenotypic resistance profiles and clinical source of isolation (blood, CSF and rectal swab). Sequence data were analysed using comparative genomics and phylogenetics.

**Results:** Our results revealed the presence of five clades, which were strongly associated with *E. coli* phylogroups A, B1, B2, D and F. We identified 43 multilocus STs, of which ST131 (14.9%) and ST12 (9.6%) were the most common. We identified 25 AMR genes. The most common ESBL gene was *bla*_CTX-M-15_ and it was present in all five phylogroups and 11 STs, and most commonly detected in ST391 (4/4 isolates), ST648 (3/3 isolates) and ST131 [3/14 (21.4%) isolates].

**Conclusions:** This study has revealed a high diversity of lineages associated with AMR, including ESBL and fluoroquinolone resistance, in Malawi. The data highlight the value of longitudinal bacteraemia surveillance coupled with detailed molecular epidemiology in all settings, including low-income settings, in describing the global epidemiology of ESBL resistance.

## Introduction

In Africa, *Escherichia coli* is the second most common Gram-negative cause of community-acquired bloodstream infection, accounting for 7.3% of all confirmed bloodstream infection isolates,^1^ and is also one of the leading causes of diarrhoeal disease, which is responsible for >10.0% of all deaths globally in children under 5 years old, and >11.0% in Africa.^2^^,^^3^*E. coli* is rapidly becoming resistant to first-line and last-resort antimicrobial agents, including cephalosporins, fluoroquinolones and, in some settings, carbapenems,^4–7^ resulting in infections that are difficult or impossible to treat depending on local availability of antimicrobials. The spread of antimicrobial resistance (AMR) has often been attributed to selective pressure resulting from increased use of antimicrobial agents.^8^ In most developing countries, the high burden of invasive bacterial infection, the high prevalence of immune-suppressive conditions (e.g. malnutrition and HIV) and lack of diagnostic microbiology facilities necessitates the widespread use of broad-spectrum antimicrobials. In Malawi, high levels of cephalosporin and fluoroquinolone resistance in *E. coli* strains have been reported following extensive use of ceftriaxone and ciprofloxacin.^9^ Cephalosporin- and fluoroquinolone-resistant *E. coli* have also been reported from a number of other countries in sub-Saharan Africa (SSA).^10–12^

The main mechanism of cephalosporin resistance is drug inactivation, which is mediated by hydrolysis of the β-lactam ring of the antimicrobial agent by ESBL enzymes. CTX-M derivatives, and in particular CTX-M-15, are now the dominant and most widely disseminated ESBL type found amongst *E. coli* isolates.^7^ Furthermore, despite the high genetic diversity associated with the *E. coli* species,^13^ ESBLs, especially of the CTX-M type, are strongly associated with specific clonal *E. coli* lineages.^8^ In the case of CTX-M-15, it is predominantly associated with a globally disseminated ST131 clone.^8^^,^^14^ The ESBL-producing lineages are also associated with fluoroquinolone resistance^15^ despite being mediated by different mechanisms. Unlike cephalosporin resistance, fluoroquinolone resistance is mainly conferred by chromosomal mutations in the quinolone-binding target genes, such as the subunits of the gyrase genes *gyrA* or *gyrB* and topoisomerase IV genes *parC* or *parE.*^16^ Plasmid-mediated genes such as *qnr*, *qep* and *aac(6ʹ)-Ib-cr* have also been shown to confer low-level fluoroquinolone resistance.^16^

WGS has emerged as an essential tool in understanding the emergence and spread of resistant strains and therefore informing strategies to combat AMR. Whilst the emergence of ESBL and fluoroquinolone resistance in SSA is a major public health concern, there are limited studies to provide genomic insight into the molecular patterns underlying the spread of ESBL and fluoroquinolone resistance in the region. To our knowledge, we carried out this first *E. coli* WGS study in Malawi to provide a baseline for understanding the genetic population structure of clinical isolates of *E. coli* and identify *E. coli* lineages associated with ESBL resistance in this setting.

## Materials and methods

### Ethics

The Malawi-Liverpool-Wellcome Trust Clinical Research Programme (MLW) routine culture surveillance, under which the isolates were obtained, was approved by the College of Medicine Research Ethics Committee (COMREC) of the University of Malawi, approval number P.08/14/1614.

### Study setting and sample collection

We used samples collected as part of routine bacteraemia and meningitis surveillance undertaken at Queen Elizabeth Central Hospital (QECH), Blantyre, Malawi, and archived at MLW. QECH is the largest government hospital in Malawi, serving Blantyre (population ∼1.3 million) and the surrounding districts. Adults had blood sampled for culture when admitted to QECH with fever (axillary temperature >37.5°C) or clinical evidence of sepsis.^17^ CSF samples were taken from the adult patients where there was clinical suspicion of meningitis.^18^ Blood or CSF samples were taken from children with febrile illness, but who were malaria slide negative and had no obvious focus of infection, or who were severely ill with sepsis or meningitis, and patients who failed initial malaria treatment and remained febrile.^19^ Antimicrobial susceptibility tests were performed by the disc diffusion method using methods and breakpoints outlined by BSAC (www.bsac.org.uk). Isolates were tested against six commonly used antimicrobial agents, namely ampicillin, co-trimoxazole, chloramphenicol, gentamicin, cefpodoxime and ciprofloxacin. Isolates were stored on beads at −80°C.

### Isolate selection and DNA extraction

Sixty blood culture and 26 CSF *E. coli* isolates were selected from the MLW archives on the basis of diversity of phenotypic resistance profiles and clinical source of isolation. Isolates originated from paediatric (<16 years old) and adult (≥16 years old) patients from the period 1996–2014. Eight rectal swab isolates, all from 2009, were also included. Whole-genome DNA extraction for selected isolates was done at MLW laboratories using the Qiagen Universal Biorobot (Limburg, the Netherlands), following the manufacturer’s instructions.

### WGS, de novo assembly and sequence annotation

Genomic DNA libraries were prepared and sequenced at the Wellcome Trust Sanger Institute (WTSI) in Hinxton, UK, using the Illumina Hiseq 2000 platform (Illumina, Inc., San Diego, CA, USA). This generated paired-end sequence reads of 100 bp. The sequence reads were trimmed and checked for quality using an average base pair quality score of at least 20 per read. We used Velvet v1.2.09,^20^ a *de novo* sequence assembly method that uses the De Bruijn graph approach to generate contiguous sequences (contigs) by selecting the optimal k-mers using VelvetOptimiser.^21^ Assembled contigs with <300 bp were excluded from the dataset and the remaining contigs were scaffolded using SSPACE Basic v2.0.^22^ The gaps between contigs were filled by GapFiller v1.10.^23^ Sequence assemblies were annotated using the Prokka v1.11 bacterial annotation pipeline.^24^ Sequence reads were deposited in the European Nucleotide Archive (ENA). Isolate accession numbers, sequencing and assembly statistics are included in Table S1 (available as Supplementary data at *JAC* Online).

### Phylogeny reconstruction and inference of population structure

The Roary pan-genome pipeline^25^ was used to construct the core genome of the 94 *E. coli* strains using the annotated genome assemblies. Genes were classified as ‘core’ if they were identified in at least 99% of the isolates. All genes present in <99% of the isolates were classified as ‘accessory’. We aligned each core gene and concatenated all the alignments to generate a single core-gene multiple sequence alignment, which was used to construct a maximum likelihood (ML) phylogenetic tree using RAxML v.7.8.6.^26^ RAxML was run under the General Time Reversible (GTR) substitution model with a Gamma rate of correction heterogeneity. The reliability of the inferred branches and branch partitions in the phylogenetic tree were assessed using 100 bootstrap replicates.

To determine the underlying genomic population structure of the study isolates, we mapped the sequence reads for all 94 isolates onto the *E. coli* strain K12 sub-strain MG1655 reference whole-genome sequence (accession number U00096) using Burrows Wheeler Aligner (BWA).^27^ Realignment of the insertion and deletion (indel) sites in the generated binary alignment map (BAM) files for the isolates was performed using GATK v3.3.0.^28^ The consensus pseudo-sequences were generated from each BAM file and aligned with other sequences to generate whole-genome alignment of the study isolates. We adjusted for recombination and used Gubbins^29^ to remove recombination sites from the whole-genome alignment and then generated an alignment of polymorphic (variable) sites only using SNP-Sites.^30^ An ML phylogenetic tree was then reconstructed using this SNP alignment, again using RAxML with the same parameters used to construct the core-gene alignment tree. Topologies of the core-gene alignment tree and mapping (to reference) alignment SNP tree were compared by a tanglegram drawn with a dendroscope.^31^ This SNP alignment was also used to cluster the isolates into unique subpopulations or sequence clusters (SCs) using the hierBAPS module in the Bayesian analysis of population structure (BAPS) v6.0 software.^32^

### In silico molecular typing of study isolates

We did *in silico* MLST^33^ and PCR to determine the STs and phylogroups of study isolates. MLST was performed using seven housekeeping genes, namely *adk*, *fumC*, *gyrB*, *icd*, *mdh*, *purA* and *recA* (http://mlst.warwick.ac.uk/mlst/dbs/Ecoli). For phylogroup typing, a two-stage *in silico* quadruplex PCR was performed as described previously by Clermont *et al.*^34^ In brief, the first stage of the PCR used four primers: *arpA*, *chuA*, *yjaA* and *TspE.C2*. If a given isolate’s phylogroup could not be resolved from the first stage, a second stage was performed that included two more primer sequences, *trpAgpC* and *arpAgpC*.

### Determination of antimicrobial resistance and plasmid typing

Acquired AMR genes were searched for by BLAST,^35^ using a database of acquired AMR genes curated at WTSI based on the ResFinder database.^36^ Presence of a gene in an isolate was confirmed if its assembled genome sequence had at least 95% nucleotide identity match with a gene in the database and a coverage of at least 90% of the length of the database match. We analysed translated nucleotide sequence alignments of the *gyrA*, *gyrB*, *parC* and *parE* genes to identify specific amino acid mutations that were associated with fluoroquinolone resistance. *In silico* plasmid typing was done by searching for plasmid replicons against the PlasmidFinder database.^37^

### Statistical analyses

Proportions were compared using Fisher’s exact test or the χ^2^ test where appropriate and means of pairwise SNP differences for identified clades were compared using one-way analysis of variance (ANOVA). We used the power-law regression equation to model the expansion of the pan-genome in relation to number of isolates. All statistical analyses were done using R v3.3.1 (R Core Team, www.r-project.org).

## Results

### Pan-genome of Malawian E. coli isolates

Ninety-four *E. coli* strains were sequenced and included for comparative genomic analysis. Automated annotation predicted an average of 4845 genes per isolate, which resulted in an overall pan-genome consisting of 22 934 genes. Genes were considered to be the same if they were identical in at least 95% of the nucleotide positions. The core gene set comprised 2210 genes, which is equivalent to 9.6% of the pan-genome size and 45.6% of the average whole genome size, while the accessory gene set consisted of 20 724 genes, i.e. 90.4% of the pan-genome size. The cumulative number of genes in the pan-genome, the number of core genes per subset of isolates, the number of isolate-specific (unique) genes per subset of isolates and the number of newly identified genes when more genomes were added to the collection were identified (Figure S1). The pan-genome analysis has shown that the *E. coli* population in Blantyre, Malawi, has an open pan-genome (Figure S2), consistent with previous studies.^38^^,^^39^ This implies that the pan-genome will continue to expand with the inclusion of more genome sequences. The majority of the newly discovered genes with an increasing number of isolates were unique (Figure S1) and approximately one-third of the total accessory genes consisted of strain-specific genes.

### Sequence types and phylogroups

**Figure 1.**
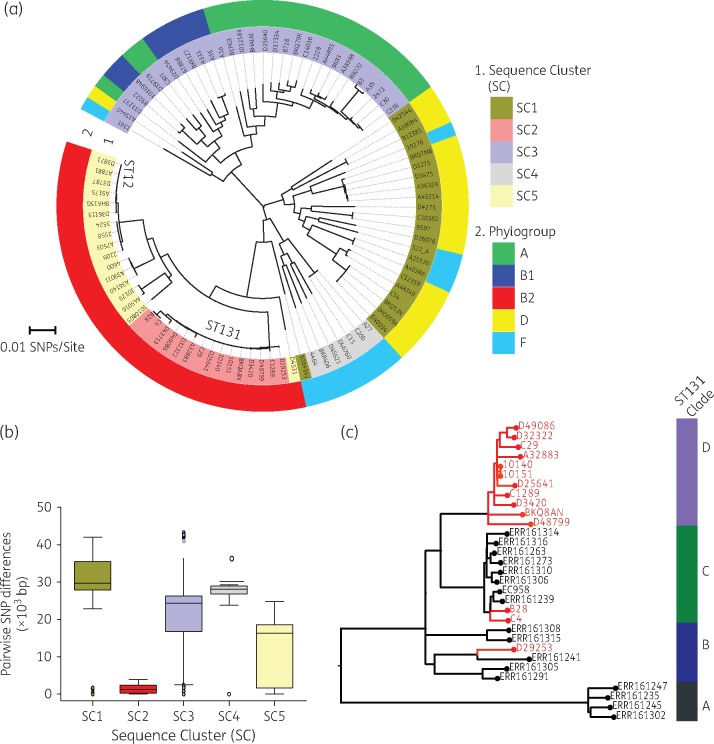
Genomic population structure and relatedness of *E. coli* isolates collected from Blantyre, Malawi. (a) Circular ML core-genome phylogenetic tree of Malawian *E. coli* isolates rooted at mid-point of the longest branch separating the two most divergent isolates. The inner ring designates identified SCs by hierBAPS and the outer ring designates phylogroups identified by *in silico* PCR. (b) Distribution of pairwise SNP differences in each of the five clades to demonstrate the variations in sequence diversity in each clade. (c) An ML phylogenetic tree, constructed from recombination-free SNP alignment of Malawian ST131 isolates (red branches) in the context of previously published global ST131 isolates (black branches). This figure appears in colour in the online version of *JAC* and in black and white in the printed version of *JAC*.

The degree of sequence diversity in the study isolates was further analysed using MLST and phylogrouping. Our results from MLST showed that the *E. coli* isolates belonged to 45 different STs. Seven of the isolates could not be uniquely typed by MLST. ST131 was the most common ST, identified in 14.9% (14/94) of the isolates, and was followed by ST12, which was present in 9.6% (9/94) of the isolates. Other common STs included ST69 (5.0%), ST10 (5.0%) and ST391 (4.0%). Overall, a total of five phylogroups, namely A, B1, B2, D and F, were identified in the study isolates. Phylogroup B2, which was predominantly composed of ST131 and ST12 isolates, was the largest phylogroup in this collection (Figure 1a), containing 34.0% (32/94) of the isolates. Phylogroup A was the second largest phylogroup with 23.4% (22/94) isolates.

### Population structure of Malawian E. coli isolates

**Figure 2.**
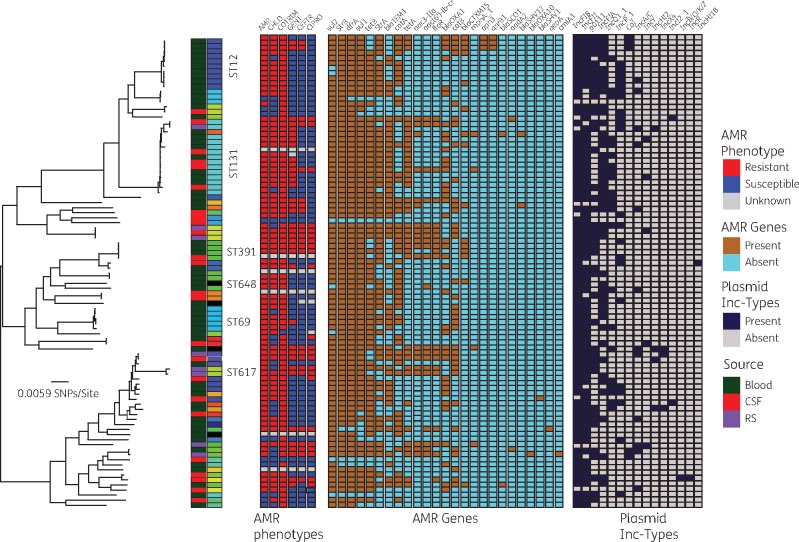
Distribution of AMR phenotype profiles, acquired AMR genes and plasmid incompatibility groups across the phylogeny of Malawian *E. coli* isolates. On the left is the ML core-genome phylogeny of the *E. coli* isolates from Malawi. The first two columns at the termini of the phylogeny represent the clinical source of isolation and STs for each isolate, respectively. Immediately following the two columns are six columns that represent the AMR phenotype profile. The next two panels following the AMR phenotype profiles are columns that represent presence and absence of AMR genes and plasmid replicons. This figure appears in colour in the online version of *JAC* and in black and white in the printed version of *JAC.*

A population structure analysis using the hierBAPS sequence-clustering approach clustered the 94 *E. coli* isolates into five clades or SCs designated SC1–SC5. The inferred SCs correlated with the *E. coli* phylogroups described in the previous section (Figure 1a). The SCs showed significant differences in nucleotide sequence diversity (*P *<* *0.001) as demonstrated by the distribution of pairwise SNP differences in each cluster (Figure 1b). The phylogeny reconstructed from the core genome was compared with that reconstructed from whole-genome sequence data adjusted for recombination, and this revealed a similar topology (Figure S3). The phylogenetic clades inferred from the core-genome ML phylogenetic tree were also consistent with the hierBAPS clusters. SC2 and SC5 showed the lowest within-cluster sequence diversity, characterized by short phylogenetic branches and lower average pairwise SNP differences of 1419 and 12 300 core-genome SNPs, respectively. In comparison, SC1, SC3 and SC4 demonstrated higher average pairwise SNP differences of 29 010, 22 230 and 23 950 core-genome SNPs, respectively (Figure 1b). The highly divergent clusters, namely SC1, SC3 and SC4, comprised diverse sets of STs, i.e. 9 STs in SC1, 23 STs in SC3 and 6 STs in SC4; the less diverse SCs, namely SC2 and SC5, on the other hand, were principally associated with ST131 and ST12. Except for SC5, which comprised blood culture isolates only (Figure 2), there was generally no clustering of isolates specific to either source of isolation or patients’ age group.

To understand the Malawian ST131 isolates in the context of other global isolates, we mapped the 14 Malawian ST131 genomes and selected 17 previously published ST131 genomes (from all three identified ST131 clades)^40^ to the ST131 EC958 reference genome and generated an ML phylogeny from recombination-free SNP alignment. Two CTX-M-15 isolates from Malawi (B28 and C4) clustered with isolates from the CTX-M-15-associated sub-clade C, whereas one CTX-M-15 isolate (BKQ8AN) clustered with the rest of the other Malawian non-CTX-M-15 isolates into a sub-clade that we have designated D, which was distinct from the previously known sub-clades A, B and C (Figure 1c).

### Genetic determinants of antimicrobial resistance in E. coli

Our analysis identified a total of 25 unique AMR genes known to encode proteins conferring resistance to a range of different classes of antimicrobial compounds, including aminoglycosides, β-lactams, chloramphenicol, sulphonamides, trimethoprim, tetracyclines and fluoroquinolones (Table 1). AMR genes, especially those present in at least four isolates, were distributed across the *E. coli* phylogeny independent of phylogenetic clustering of the host isolates (Figure 2). Similar to phylogenetic clustering, there were no specific AMR genotypes associated with isolates from a particular source or patient age group.

**Table 1.** Distribution of AMR genes among Malawian *E. coli* isolates

Gene	Known AMR phenotype	Isolates, *n* (%)
*sul2*	sulphonamides	88 (93.6)
*strA*	aminoglycosides	87 (92.6)
*strB*	aminoglycosides	87 (92.6)
*dfrA*	trimethoprim	86 (91.5)
*bla*_TEM-1_	β-lactams	74 (78.7)
*catA*	chloramphenicol	61 (64.9)
*sul1*	sulphonamides	51 (54.3)
*tetB*	tetracycline	51 (54.3)
*aac3-IIa*	aminoglycosides	37 (39.4)
*tetA*	tetracycline	38 (40.4)
*mphA*	macrolides	35 (37.2)
*bla*_CTX-M-15_	ESBL	20 (21.3)
*bla*_OXA-1_	β-lactams	20 (21.3)
*aac(6ʹ)-Ib-cr*	aminoglycosides, fluoroquinolone	18 (19.1)
*aph3*	aminoglycosides	15 (16.0)
*qnrB1*	fluoroquinolones	4 (4.3)
*floR*	chloramphenicol	3 (3.2)
*bla*_SCO-1_	β-lactams	2 (2.1)
*aadA2*	aminoglycosides	2 (2.1)
*bla*_SHV-12_	ESBL	2 (2.1)
*catB*	chloramphenicol	2 (2.1)
*cmlA1*	chloramphenicol	1 (1.1)
*bla*_OXA-10_	ESBL	1 (1.1)
*bla*_SHV-1_	β-lactam	1 (1.1)
*qepA*	quinolones	1 (1.1)

### β-Lactam and ESBL resistance

We identified seven genes associated with β-lactam resistance, including three ESBL genes (Table 1). Narrow-spectrum β-lactamases included *bla*_TEM-1_ (74/94 isolates), *bla*_OXA-1_ (20/94 isolates), *bla*_SCO-1_ (2/94 isolates) and *bla*_SHV-1_ (1/94 isolates). Twenty-one out of 94 (22.3%) isolates carried at least one ESBL gene, of which *bla*_CTX-M-15_ was the most common (Table 1), being present in 20/21 (95.2%) of the isolates carrying an ESBL gene. The *bla*_CTX-M-15_ gene was significantly associated with resistance to cephalosporins (*P *<* *0.001), with 19/20 isolates carrying the *bla*_-CTX-M-15_ gene being resistant to ceftriaxone by disc testing. The ceftriaxone resistance phenotype was not known for the remaining isolate with the *bla*_CTX-M15_ gene. Other ESBLs detected included *bla*_SHV-12_ (one isolate) and *bla*_OXA-10_ (one isolate), and both isolates had expressed phenotypic resistance to ceftriaxone. Similar to all common AMR genes identified in this collection, the *bla*_CTX-M-15_ gene was present in isolates independent of phylogenetic clustering. The distribution of isolates carrying *bla*_CTX-M-15_ showed that the gene was present in isolates from five identified phylogroups and 11 STs, most commonly ST391 (4/4 isolates), ST648 (3/3 isolates) and ST131 [3/14 (21.4%) isolates] (Table 2). The *bla*_CTX-M-15_ genes were found to be located in both the chromosome (5/20 isolates) and plasmid DNA (15/20 isolates), but their location was always downstream and adjacent to the IS*Ecp1* element.

**Table 2.** Characteristics of Malawian CTX-M-15-associated *E. coli* isolates

Isolate ID	Year of isolation	Source	ST	Phylogroup	Genome locus	Plasmid replicon
D42544	2007	blood	1084	D	plasmid	FIB, FII, HI2
BKQA8N	2013	blood	131	B2	chromosome	FIB, FII, HI2, P, Q1
C4	—	CSF	131	B2	chromosome	Col, FIA, FIB, Q1
B28	2009	RS	131	B2	chromosome	Col, FIA, FII, X4, Y
C30	2009	RS	167	A	plasmid	FIA, FIB, FII, I2, Y
C33b	—	CSF	167	A	plasmid	FIA, FIB, FII, I2, P, Y
1012184	2011	blood	361	A	plasmid	FIB, FII
D40034	2006	blood	391	D	plasmid	FIB, FIA, X1
BKQ5JN	2013	blood	391	D	plasmid	FIB, FIA, X1, X4
D40059A	2006	blood	391	D	plasmid	FIA, FIB, X1
C14	—	CSF	391	D	plasmid	A/C, FIA, FIB, FII, Q1
A1a	2009	RS	399	A	plasmid	HI2, Y
A16	2009	blood	448	B1	plasmid	A/C, FIA, FIB, FII, Q1
A3b	2009	RS	617	A	plasmid	FIA, FIB, FII
A27	2009	RS	648	F	chromosome	FIA, FIB, FII
C15	—	CSF	648	F	chromosome	FIA, FIB, FII
C20b	2009	RS	648	F	chromosome	Col, FIA, FIB, FII
1016948	2011	CSF	977	B1	plasmid	Y
1014142	2011	blood	1163	F	plasmid	FIA, FIB, FII, Q1, Y

RS, rectal swab.

### Fluoroquinolone resistance

We translated the aligned nucleotide sequences of each gene into protein sequences. The amino acid products of the *gyrA* gene alignment were highly conserved. However, we identified amino acid substitutions at codon position 83 from serine (S) to leucine (L), (S83L), in 17.0% (16/94) and serine (S) to alanine (A), (S83A), in 2.1% (2/94) of the isolates, and also at codon position 87, which showed a change from aspartic acid (D) to asparagine (N), (D87N), in 14.9% (14/94) of the isolates. Fourteen isolates had mutations at both codon positions 83 and 87 and all of them showed i*n vitro* resistance to ciprofloxacin, whereas two isolates had only one codon mutation at position 83 and they were fluoroquinolone susceptible. In addition to the identified fluoroquinolone mutations, we also identified four isolates carrying the horizontally acquired plasmid-mediated fluoroquinolone resistance gene *qnrB*. None of the four isolates carrying the *qnrB* gene had the *gyrA* fluoroquinolone resistance mutations, but three isolates showed reduced susceptibility to fluoroquinolones.

### Aminoglycoside resistance

Six genes known to confer resistance to aminoglycosides were identified in this dataset. These include *strA* and *strB* in 87/94 (92.6%) isolates, *aac3-IIa* in 37/94 (39.4%) isolates, *aac(6′)-Ib-cr* in 18/94 (19.1%) isolates, *aph3* in 15/94 (16.0%) isolates and *aadA* in 2/94 (2.1%) isolates (Table 1). Both *aac3-IIa* and *aac(6′)-Ib-cr* were associated with gentamicin as well as fluoroquinolone resistance phenotypes (*P *<* *0.001). Together, *strA* and *strB* are known to confer resistance to streptomycin, which is only used in Malawi for re-treatment of TB, and there was no significant association between these genes and gentamicin resistance phenotype (*P *=* *0.318).

### Chloramphenicol resistance

Four genes known to encode chloramphenicol resistance were identified: *catA* in 61/94 (64.9%) isolates; *floR* in 3/94 (3.2%) isolates; *catB* in 2/94 (2.1%) isolates; and *cmlA1* in 1/94 (1.1%) isolates (Table 1). The *in silico* predictions were confirmed by phenotypic resistance data, with chloramphenicol resistance being strongly associated with the presence of the *catA* gene (*P *<* *0.001). All three isolates carrying the *floR* gene had a chloramphenicol resistance phenotype, whereas the isolate with the *cmlA1* gene was susceptible to chloramphenicol. The isolate with the *catB* gene also had the *catA* gene and was chloramphenicol resistant.

### Plasmid incompatibility groups

A search against the PlasmidFinder database identified a total of 15 different plasmid replicons (including FIA, FIB, FII, Col, A/C, HI1B, HI2, B/O/K/Z, I2, Q, P, p0111, R, X4 and Y) to be present in our collection of *E. coli* isolates (Figure 2). FIB, FIA and FII were the most common plasmid replicons across all the 94 isolates (Figure 2) but also amongst isolates that had the *bla*_CTX-M-15_ gene (Table 2). One IncFIB plasmid was similar to pNDM-Mar1 (GenBank accession number JN420336), a plasmid known to encode the carbapenem resistance gene *bla*_NDM-1_, although no carbapenem resistance gene was identified in this dataset.

## Discussion

*E. coli* is well known to be a highly diverse species^13^ that is strongly associated with AMR. In this study, we whole-genome sequenced 94 *E. coli* isolates collected in Blantyre, Malawi, situated in a region from which such data are severely lacking. We reveal that *E. coli* in this setting has an expanding pan-genome, and high diversity in phylogenetic clustering, STs and phylogroups consistent with the known global diversity. Furthermore, we demonstrate that AMR, in particular ESBL resistance, in Malawi has emerged across a diverse range of *E. coli* lineages containing a number of similar resistance genes.

The most commonly identified ESBL gene was *bla*_CTX-M-15_, present in 11 STs and all five identified phylogroups, including all ST391 genomes, 3/14 ST131 genomes and a number of other clonally diverse STs (Table 2). Within SSA, a high diversity of CTX-M-15-associated STs has also been reported in Tanzania,^11^ whereas globally the *bla*_CTX-M-15_ gene has been more strongly associated with a sub-lineage of ST131, *H30*-Rx.^41^ The predominance of this ST131 sub-lineage as a CTX-M-15 clone has been reported in Asia, Europe, North Africa and South America.^7^^,^^42–45^ Further work is required in Malawi to accurately estimate the prevalence of *bla*_CTX-M-15_ across the different clades causing drug-resistant infection.

ST131 isolates in this study constitute the most clonal SC in the population structure of our collection, suggesting that they were the most recent lineage to emerge or arrive in this setting. A previous study of a global collection of ST131 isolates identified three sub-lineages of the ST131 isolates, which were designated A, B and C.^40^ When put in the context of these global ST131 isolates, the majority of the Malawian ST131 isolates form a sub-lineage distinct from these previously identified sub-lineages (Figure 1c). Furthermore, whilst two of the three CTX-M-15 isolates from Malawi cluster in the CTX-M-15-associated sub-lineage C, one clusters with the rest of the other Malawian isolates in sub-clade D. The CTX-M-15-associated ST131 clone is reported to have emerged in the early to mid 2000s^7^ and has since spread in a series of clonal expansions to become the most dominant *E. coli* clone, causing a spectrum of extra-intestinal infections, including urinary tract infections, bacteraemia, pneumonia, and intra-abdominal and wound infections.^44^ In our collection, the earliest CTX-M-15-associated ST131 isolate was isolated in 2009, and although the CTX-M-15 ST131 clone might have emerged earlier in this setting, a previous study of ESBL-producing Enterobacteriaceae in 2004–05 did not identify CTX-M-15-producing *E. coli*.^46^ It is therefore likely that CTX-M-15-associated ST131 emerged or arrived in this setting a few years after its emergence in Europe and North America.

In this study, we have further shown that the *bla*_CTX-M-15_ gene exists in genetically heterogeneous strains even within the ST131 lineage (Figure 1a and c). It is not only the case that *bla*_CTX-M-15_ was distributed across phylogenetically diverse isolates, but fluoroquinolone resistance genotypes and all other common AMR genes, including *dfrA*, *sul1*, *sul2*, *strA*, *strB*, *bla*_TEM-1_, *catA* and *tetB*, were also widely distributed. The set of common AMR genes, namely *dfrA*, *sul1*, *sul2*, *strA*, *strB*, *bla_TEM-1_*, *catA* and *tetB*, has previously been characterized in MDR *Salmonella* circulating in Blantyre but, unlike with *E. coli*, these were associated with epidemic clades.^47^^,^^48^

In conclusion, this study has revealed a high diversity of lineages of *E. coli* circulating in Malawi and associated with AMR, including ESBL and fluoroquinolone resistance. Further work is required to ascertain which clades are the predominant cause of drug-resistant infection in this setting. The data highlight the value of longitudinal bacteraemia surveillance coupled with detailed molecular epidemiology in all settings in describing the global epidemiology of ESBL resistance.

## Supplementary Material

Supplementary Data
