# Supplementary material for: Genomic landscape of extended-spectrum β-lactamase resistance in Escherichia coli from an urban African setting
Source: J Antimicrob Chemother. 2017 Mar 4;72(6):1602–9. doi: 10.1093/jac/dkx058 (PMC5437524; doi:10.1093/jac/dkx058)
Supplement: Supplementary Data [file dkx058_Supp.docx]

**Supplementary data**

**Figure S1** **Distributions of genes in the *E. coli* isolates collected from Blantyre, Malawi.** Box plots showing distribution of number of genes in 10 samples obtained iteratively for a given number of genome sequences.  **(A)** Shows total number of single copy genes identified in the pan-genome of given set of *E. coli* genome sequences. **(B)** Shows total number of conserved (core) genes identified in at least 99% of a given set of genome sequences. **(C)** Shows number of ‘new’ genes identified per added genome sequence **(D)** Shows the number of genes unique genes i.e. identified in only one isolate as the number of *E. coli* isolates increased.

**Figure S2 Expansion of the pan-genome size with increasing number of genome sequences as modelled by the power law-regression equation**$F\left( N \right)=\beta N^{\gamma}$ $F\left( N \right)=\beta N^{\gamma}$ **illustrating an open pan-genome of the *E. coli* isolates.** By normalising the number of genes obtained in the 10 iterative samples per given set of genome sequences as described in Figure S1, the values of β and γ were estimated to obtain the power-law regression equation $F\left( N \right)=e^{8.4446}N^{0.3551}$ $F\left( N \right)=e^{8.4446}N^{0.3551}$depicted by the red line-graph. The points in black represent the average number of genes in the pan-genome of a given set of genomes.

Figure S3 Tanglegram comparing maximum likelihood (ML) phylogeny based on core genome alignment (right) with ML phylogeny based on whole genome sequence SNP alignment adjusted for recombination (left), revealing similar topology.

Table S1: European Nucleotide Accession Numbers and assembly statistics for sequenced isolates (RS=rectal swab).

| **Strain**  **ID** | **ENA AccessionID** | **Source** | **Country** | **Total Length** | **No. of contigs** | **Avg. Contig Length** | **N50** |
| --- | --- | --- | --- | --- | --- | --- | --- |
| A39011 | ERS668966 | Blood | Malawi | 5273808 | 99 | 53270.79 | 205901 |
| 522_A | ERS668975 | Blood | Malawi | 5461868 | 86 | 63510.09 | 205687 |
| A45214 | ERS668976 | Blood | Malawi | 5284968 | 96 | 55051.75 | 175071 |
| 1010805 | ERS668977 | Blood | Malawi | 5204957 | 176 | 29573.62 | 87988 |
| BHA15G | ERS668978 | Blood | Malawi | 5290632 | 80 | 66132.9 | 313280 |
| BKQ7M8 | ERS668979 | Blood | Malawi | 5312488 | 123 | 43190.96 | 160639 |
| BKR1Z7 | ERS668980 | CSF | Malawi | 4903006 | 57 | 86017.65 | 235975 |
| D40034 | ERS668981 | Blood | Malawi | 5336252 | 102 | 52316.2 | 181853 |
| BKQ5JN | ERS668982 | Blood | Malawi | 5380861 | 82 | 65620.26 | 177456 |
| A7898 | ERS668983 | Blood | Malawi | 5480078 | 181 | 30276.67 | 127272 |
| 3361 | ERS668984 | CSF | Malawi | 5503294 | 139 | 39592.04 | 280302 |
| D3787 | ERS668967 | Blood | Malawi | 5387283 | 162 | 33254.83 | 150578 |
| BKR406 | ERS668987 | CSF | Malawi | 5164557 | 168 | 30741.41 | 84814 |
| D3475 | ERS668988 | Blood | Malawi | 5283543 | 109 | 48472.87 | 126471 |
| B12381 | ERS668989 | CSF | Malawi | 5112808 | 125 | 40902.46 | 127923 |
| A7503 | ERS668990 | Blood | Malawi | 5326194 | 198 | 26899.97 | 156280 |
| C301 | ERS668991 | CSF | Malawi | 5015203 | 95 | 52791.61 | 147873 |
| A38084 | ERS668992 | Blood | Malawi | 5108748 | 104 | 49122.58 | 139415 |
| D3275 | ERS668993 | Blood | Malawi | 5270756 | 90 | 58563.96 | 180266 |
| A5175 | ERS668994 | Blood | Malawi | 5294462 | 132 | 40109.56 | 180364 |
| B1PG3 | ERS668968 | Blood | Malawi | 5172184 | 158 | 32735.34 | 87822 |
| D4531 | ERS668995 | Blood | Malawi | 4953068 | 114 | 43447.96 | 121138 |
| 2473 | ERS668996 | Blood | Malawi | 4919531 | 84 | 58565.85 | 210321 |
| D3420 | ERS668998 | Blood | Malawi | 5457019 | 156 | 34980.89 | 188255 |
| C10382 | ERS668999 | CSF | Malawi | 5319183 | 123 | 43245.39 | 131769 |
| 2228 | ERS669000 | CSF | Malawi | 5070388 | 155 | 32712.18 | 108237 |
| 10151 | ERS669001 | CSF | Malawi | 5350547 | 101 | 52975.71 | 189034 |
| 8728 | ERS669002 | CSF | Malawi | 4897747 | 147 | 33318.01 | 85461 |
| 10129 | ERS669004 | CSF | Malawi | 4951467 | 68 | 72815.69 | 261762 |
| A36329 | ERS669005 | Blood | Malawi | 5362879 | 106 | 50593.2 | 165106 |
| C1289 | ERS668969 | CSF | Malawi | 5276857 | 145 | 36392.12 | 221661 |
| B9070 | ERS669006 | CSF | Malawi | 4885674 | 104 | 46977.63 | 150391 |
| 9597 | ERS669007 | CSF | Malawi | 5094548 | 55 | 92628.15 | 206791 |
| D36115 | ERS669008 | Blood | Malawi | 5213958 | 122 | 42737.36 | 265285 |
| 2209 | ERS669009 | Blood | Malawi | 5289536 | 102 | 51858.2 | 184080 |
| A25576 | ERS669010 | Blood | Malawi | 5428549 | 171 | 31745.9 | 129069 |
| D37334 | ERS669011 | Blood | Malawi | 5021537 | 200 | 25107.69 | 78986 |
| D25640 | ERS669012 | Blood | Malawi | 5282620 | 166 | 31823.01 | 143794 |
| D25641 | ERS669013 | Blood | Malawi | 5346456 | 94 | 56877.19 | 191459 |
| D29454 | ERS669014 | Blood | Malawi | 5113300 | 115 | 44463.48 | 183101 |
| A38988 | ERS669015 | Blood | Malawi | 5382272 | 208 | 25876.31 | 108438 |
| 1012184 | ERS668970 | Blood | Malawi | 4938419 | 95 | 51983.36 | 118104 |
| A40286 | ERS669016 | Blood | Malawi | 5587432 | 288 | 19400.81 | 93942 |
| 4464 | ERS669017 | CSF | Malawi | 5179118 | 145 | 35718.06 | 109887 |
| B9222 | ERS669018 | CSF | Malawi | 5063584 | 87 | 58202.11 | 240689 |
| C14036 | ERS669019 | CSF | Malawi | 5244671 | 193 | 27174.46 | 87604 |
| A36140 | ERS669020 | Blood | Malawi | 5101616 | 100 | 51016.16 | 247618 |
| 3524 | ERS669021 | Blood | Malawi | 5170502 | 109 | 47435.8 | 190806 |
| 4600 | ERS669022 | Blood | Malawi | 5394345 | 107 | 50414.44 | 215073 |
| C12359 | ERS669024 | CSF | Malawi | 5513173 | 206 | 26762.98 | 152004 |
| A32883 | ERS669025 | Blood | Malawi | 5301863 | 152 | 34880.68 | 164400 |
| BKQA8N | ERS668971 | Blood | Malawi | 5609738 | 130 | 43151.83 | 182674 |
| 9693 | ERS669027 | Blood | Malawi | 5224453 | 159 | 32858.19 | 114505 |
| 2558 | ERS669028 | Blood | Malawi | 5213209 | 162 | 32180.3 | 141239 |
| A27 | ERS669029 | RS | Malawi | 5334528 | 110 | 48495.71 | 175780 |
| C29 | ERS669031 | CSF | Malawi | 5271826 | 90 | 58575.84 | 191186 |
| A333 | ERS669032 | Blood | Malawi | 4981825 | 100 | 49818.25 | 154051 |
| D39719 | ERS669033 | Blood | Malawi | 5163387 | 93 | 55520.29 | 226049 |
| C30 | ERS669034 | RS | Malawi | 5185692 | 82 | 63240.15 | 201161 |
| C15 | ERS669035 | CSF | Malawi | 5331258 | 129 | 41327.58 | 219011 |
| D40059A | ERS669036 | Blood | Malawi | 5341539 | 92 | 58060.21 | 155469 |
| BHAIAI | ERS668972 | Blood | Malawi | 4854690 | 92 | 52768.37 | 122489 |
| D43713 | ERS669037 | Blood | Malawi | 5341172 | 98 | 54501.76 | 162589 |
| C4 | ERS669039 | CSF | Malawi | 5291658 | 126 | 41997.29 | 181330 |
| A16 | ERS669041 | RS | Malawi | 5250580 | 132 | 39777.12 | 113555 |
| A35440 | ERS669042 | Blood | Malawi | 5153397 | 219 | 23531.49 | 65279 |
| C33B | ERS669044 | CSF | Malawi | 5303054 | 122 | 43467.66 | 122520 |
| D32322 | ERS669045 | Blood | Malawi | 5588592 | 119 | 46962.96 | 189956 |
| C14 | ERS669047 | CSF | Malawi | 5329072 | 105 | 50753.07 | 166888 |
| D3871 | ERS669048 | Blood | Malawi | 5431645 | 159 | 34161.29 | 149291 |
| 1014142 | ERS669049 | Blood | Malawi | 5062819 | 120 | 42190.16 | 134448 |
| D29253 | ERS668973 | Blood | Malawi | 5440270 | 97 | 56085.26 | 203704 |
| B28 | ERS669050 | RS | Malawi | 5404547 | 88 | 61415.31 | 192412 |
| C20b | ERS669051 | RS | Malawi | 5339050 | 147 | 36320.07 | 135868 |
| D49086 | ERS669052 | Blood | Malawi | 5431220 | 129 | 42102.48 | 160139 |
| D48799 | ERS669054 | Blood | Malawi | 5232386 | 83 | 63040.8 | 204504 |
| B3 | ERS669055 | RS | Malawi | 4959972 | 100 | 49599.72 | 150867 |
| 1016948 | ERS668974 | CSF | Malawi | 5155016 | 87 | 59253.06 | 244350 |
| BKQ79K_1 | ERS669067 | Blood | Malawi | 5020018 | 210 | 23904.85 | 78702 |
| 10276 | ERS669087 | Blood | Malawi | 5281674 | 105 | 50301.66 | 211874 |
| D26076 | ERS669089 | Blood | Malawi | 5477833 | 101 | 54235.97 | 170807 |
| A7881 | ERS669101 | Blood | Malawi | 5367711 | 123 | 43639.93 | 181395 |
| D45621 | ERS669107 | Blood | Malawi | 5206131 | 62 | 83969.85 | 326072 |
| D4275 | ERS669114 | Blood | Malawi | 5030609 | 50 | 100612.18 | 260601 |
| A44893 | ERS669128 | Blood | Malawi | 5219699 | 191 | 27328.27 | 106000 |
| A1a | ERS669146 | RS | Malawi | 5201081 | 167 | 31144.2 | 84058 |
| 10140 | ERS668997 | CSF | Malawi | 5348508 | 100 | 53485.08 | 180964 |
| D42544 | ERS669030 | Blood | Malawi | 5556477 | 94 | 59111.46 | 188605 |
| D46760 | ERS669038 | Blood | Malawi | 5783150 | 192 | 30120.57 | 108336 |
| A48349 | ERS669040 | Blood | Malawi | 5361198 | 97 | 55270.08 | 175891 |
| A45016 | ERS669046 | Blood | Malawi | 4972999 | 78 | 63756.4 | 302059 |
| A3b | ERS669056 | RS | Malawi | 4937459 | 85 | 58087.75 | 143228 |
| D33237 | ERS669057 | Blood | Malawi | 4713852 | 282 | 16715.79 | 47506 |
